# Supplementary material for: Fluroxypyr Inhibits Maize Growth by Disturbing the Diversity of the Endophytic Bacterial Communities in Maize Roots
Source: Microorganisms. 2025 Mar 24;13(4):728. doi: 10.3390/microorganisms13040728 (PMC12029718; doi:10.3390/microorganisms13040728)
Supplement: Supplementary file 1 [file microorganisms-13-00728-s001.zip › microorganisms-3402130-supplementary.pdf]

microorganisms

MDPI

Supplementary

Fluroxypyr Inhibits Maize Growth by Disturbing the Diversity of the Endophytic Bacterial Communities in Maize Roots

Gangrui Zhang, Nan Liu, Shengbo Shi, Jinghua Li, Rui Geng, Longyu Fang, Yuanyuan Wang, Mingchun Lin, Junfeng Chen, Yanru Si, Kai Shan, Zeyun Zhou, Maoyu Men, Xiangren Qiao and Lujiang Hao *

School of Bioengineering, Qilu University of Technology (Shandong Academy of Sciences), Jinan 250353, China; 10431221312@stu.qlu.edu.cn (G.Z.); 10431221234@stu.qlu.edu.cn (N.L.); 10431230730@stu.qlu.edu.cn (S.S.); 10431230807@stu.qlu.edu.cn (J.L.); 10431230774@stu.qlu.edu.cn (R.G.); 10431230825@stu.qlu.edu.cn (L.F.); 10431230832@stu.qlu.edu.cn (Y.W.); 10431240798@stu.qlu.edu.cn (M.L.); 10431240800@stu.qlu.edu.cn (J.C.); 10431240837@stu.qlu.edu.cn (Y.S.); kai_shan2017@163.com (K.S.); 202281010041@stu.qlu.edu.cn (Z.Z.); 202381013075@stu.qlu.edu.cn (M.M.); 202496063019@stu.qlu.edu.cn (X.Q.)

* Correspondence: lujiang_hao@qlu.edu.cn

1. Supporting Tables

Table S1. Samples information.

Number	Treatment	Collection period	Collection Time
NR1CK	-	Seedling stage	2016.6.24
NR2	+	Flowering stage	2016.8.24
NR2CK	-	Flowering stage	2016.8.24
NR3	+	Maturation stage	2016.9.17
NR3CK	-	Maturation stage	2016.9.17
R1CK	+	Seedling stage	2016.6.24
R2	-	Flowering stage	2016.8.24
R2CK	+	Flowering stage	2016.8.24
R3	-	Maturation stage	2016.9.17
R3CK	+	Maturation stage	2016.9.17
E1CK	-	Seedling stage	2016.6.24
E2	+	Flowering stage	2016.8.24
E2CK	-	Flowering stage	2016.8.24
E3	+	Maturation stage	2016.9.17
E3CK	-	Maturation stage	2016.9.17

2. Supporting Figure

 https://doi.org/10.3390/microorganisms13040728

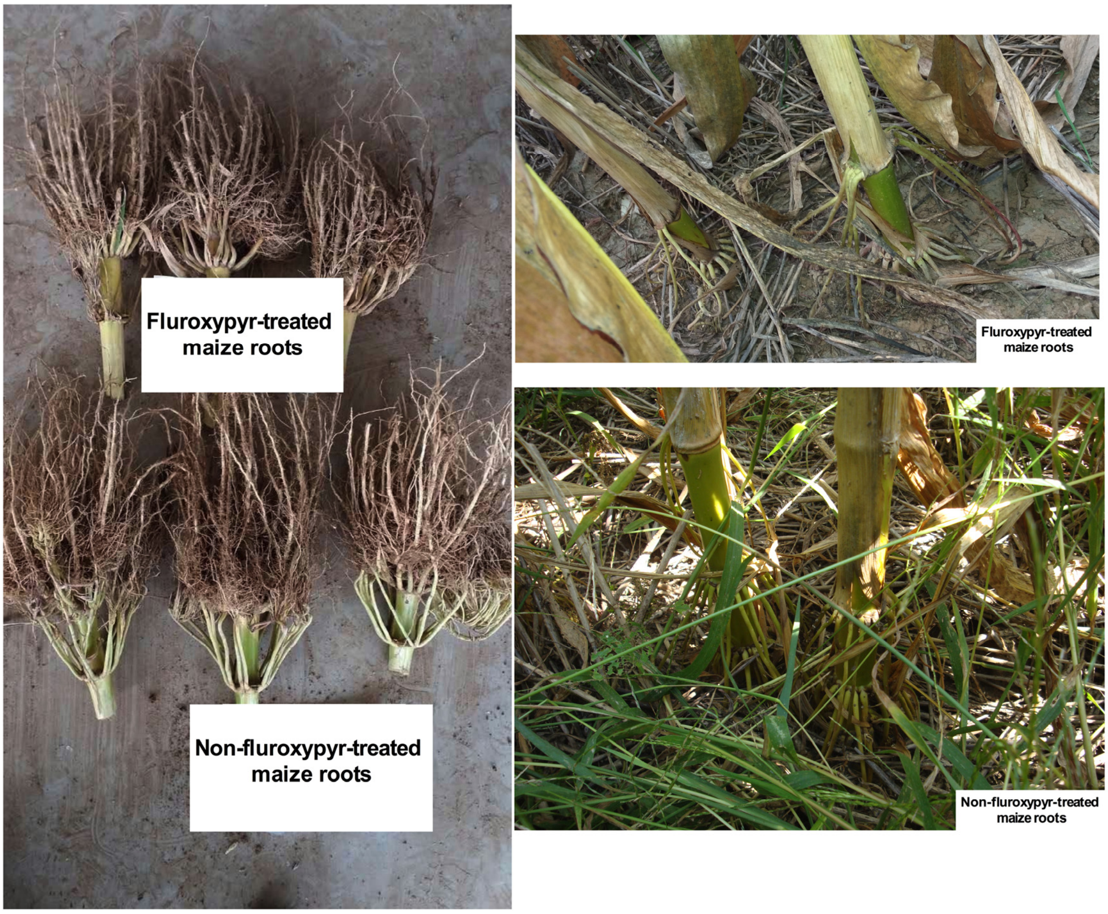

Figure S1. Corn root sample .

3. Supporting Methods

3.1. *16S rRNA gene sequencing and analysis*

3.1.1. PCR and 16 rRNA gene sequencing

The bacterial genomic DNA of soil samples was extracted using Fast DNA SPIN Kit (MP Biomedicals, Santa Ana, CA). The quantity and quality of extracted DNA was measured by a spectrophotometer (NanoDrop 1000, ThermoScientific, USA) and gel electrophoresis, re- spectively. The extracted DNA was amplified with primer 338 F (5′-ACTCCTACGGGAGGCAGCA-3′) and 806R (5′-GGACTACHVGGGT- WTCTAAT-3′), which targeted V4 region of bacterial 16S rRNA. Sample-specific 7-bp barcodes were fused to the primers.

PCR amplification of the bacterial 16S rRNA genes V3 - V4 region was performed using the universal primer set 338F/806R[1]. Sample-specific 7-bp barcodes were incorporated into the primers. Each 25 μL reaction contained 5 μL of Q5 reaction buffer (5×), 5 μL of Q5 High-Fidelity GC buffer (5×), 0.25 μL of Q5 High-Fidelity DNA Polymerase (5U / μl), 2 μL (2.5 mM) of dNTPs, 1 μL (10 μM) of each Forward and Reverse primer, 2 μL of DNA template, and 8.75 μL of ddH_2O. Thermal cycling consisted of initial denaturation at 98°C for 2 min, followed by 25 cycles of denaturation at 98°C for 15 s, annealing at 55°C for 30 s, and elongation at 72°C for 30 s, with a final extension of 5 min at 72°C. PCR amplifications were purified with Agencourt AMPure Beads (Beckman Coulter, Indianapolis, IN) and quantified using the PicoGreen dsDNA Assay Kit (Invitrogen, Carlsbad, CA, USA). After the individual quantification step, amplicons were pooled in equal amounts, and pair-end 2 × 300 bp sequencing was performed using the Illlumina MiSeq

platform with MiSeq Reagent Kit v3 at Shanghai Personal Biotechnology Co., Ltd (Shanghai, China).

3.1.2. Sequence Analysis

The Quantitative Insights Into Microbial Ecology (QIIME, v1.8.0) pipeline was employed to process the raw sequencing data[2]. Briefly, raw sequencing reads with exact matches to the barcodes were assigned to respective samples and identified as valid sequences. Reads which had ambiguous bases, mononucleotide repeats of >8 bp, a length of < 150 bp, and a quality score < 20 were discarded[3,4]. Paired-end reads were assembled using FLASH[5]. After chimera detection, the remaining high-quality sequences were clustered into operational taxonomic units (OTUs) at 97% sequence identity by UCLUST[6]. A representative sequence was selected from each OTU using default parameters. OTU taxonomic classification was conducted by BLAST searching the representative sequences set against the Greengenes Database using the best hit. An OTU table was further generated to record the abundance of each OTU in each sample and the taxonomy of these OTUs. OTUs containing less than 0.001% of total sequences across all samples were discarded.

3.1.3. Bioinformatics and Statistical Analysis

Sequence data analyses were mainly performed using QIIME and R packages (v3.2.0). Alpha diversity indices, such as Chao1, Shannon and Simpson were calculated with QIIME. OTU-level ranked abundance curves were generated to compare the richness and evenness of OTUs among samples. Beta diversity using weighted UniFrac distance metrics was calculated with QIIME[7,8]. Nonmetric multidimensional scaling (NMDS) was conducted to visualize differences in the OTU-based community composition using the 'metaMDS' function of the vegan package[9]. The significance of differentiation of bacterial structure among groups was assessed by PERMANOVA (Permutational multivariate analysis of variance)[10]and ANOSIM (Analysis of similarities)[11] using the 'adonis' function in the vegan package. Taxa abundances at the phylum, class, order, family, genus and species levels were statistically compared among samples or groups by Metastats[12]. Random forest analysis was applied to discriminating the samples from different groups using the R package "RandomForest" with 1,000 trees and all default settings. The generalization error was estimated using 10-fold cross-validation. The expected "baseline" error was also included, which was obtained by a classifier that simply predicts the most common category label[13,14].

References

1. A. Liaw, M. Wiener, Classification and regression by random forest, R News. 23 (2002). https://doi.org/doi:http://dx.doi.org/.
2. A. Ramette, Multivariate analyses in microbial ecology, FEMS Microbiol. Ecol. 62 (2007) 142–160. https://doi.org/10.1111/j.1574-6941.2007.00375.x.
3. B.H. McArdle, M.J. Anderson, Fitting multivariate models to community data: A comment on distance-based redundancy analysis, Ecology. 82 (2001) 290–297. https://doi.org/10.1890/0012-9658(2001)082[0290:FMMTCD]2.0.CO;2.
4. C. Lozupone, R. Knight, UniFrac: a new phylogenetic method for comparing microbial communities, Appl. Environ. Microbiol. 71 (2005) 8228–8235. https://doi.org/10.1128/AEM.71.12.8228-8235.2005.
5. C.A. Lozupone, M. Hamady, S.T. Kelley, R. Knight, Quantitative and aualitative β diversity measures lead to different Insights into factors that structure microbial communities, Appl. Environ. Microbiol. 73 (2007) 1576–1585. https://doi.org/10.1128/AEM.01996-06.
6. D.I. Warton, S.T. Wright, Y. Wang, Distance-based multivariate analyses confound location and dispersion effects, Methods Ecol. Evol. 3 (2012) 89–101. https://doi.org/10.1111/j.2041-210X.2011.00127.x.
7. H. Chen, W. Jiang, Application of high-throughput sequencing in understanding human oral microbiome related with health and disease, Front. Microbiol. 5 (2014). https://doi.org/10.3389/fmicb.2014.00508.

8. J. Zhang, N. Zhang, Y.-X. Liu, X. Zhang, B. Hu, Y. Qin, H. Xu, H. Wang, X. Guo, J. Qian, W. Wang, P. Zhang, T. Jin, C. Chu, Y. Bai, Root microbiota shift in rice correlates with resident time in the field and developmental stage, Sci. China Life Sci. 61 (2018) 613–621. https://doi.org/10.1007/s11427-018-9284-4.
9. J.G. Caporaso, J. Kuczynski, J. Stombaugh, K. Bittinger, F.D. Bushman, E.K. Costello, N. Fierer, A.G. Peña, J.K. Goodrich, J.I. Gordon, G.A. Huttley, S.T. Kelley, D. Knights, J.E. Koenig, R.E. Ley, C.A. Lozupone, D. McDonald, B.D. Muegge, M. Pirrung, J. Reeder, J.R. Sevinsky, P.J. Turnbaugh, W.A. Walters, J. Widmann, T. Yatsunenko, J. Zaneveld, R. Knight, QIIME allows analysis of high-throughput community sequencing data, Nat. Methods. 7 (2010) 335–336. https://doi.org/10.1038/nmeth.f.303.
10. J.R. White, N. Nagarajan, M. Pop, Statistical methods for detecting differentially abundant features in clinical metagenomic samples, PLOS Comput. Biol. 5 (2009) e1000352. https://doi.org/10.1371/journal.pcbi.1000352.
11. R.C. Edgar, Search and clustering orders of magnitude faster than BLAST, Bioinformatics. 26 (2010) 2460–2461. https://doi.org/10.1093/bioinformatics/btq461.
12. S.R. Gill, M. Pop, R.T. DeBoy, P.B. Eckburg, P.J. Turnbaugh, B.S. Samuel, J.I. Gordon, D.A. Relman, C.M. Fraser-Liggett, K.E. Nelson, Metagenomic analysis of the human distal gut Microbiome, Science. 312 (2006) 1355–1359. https://doi.org/10.1126/science.1124234.
13. T. Magoč, S.L. Salzberg, FLASH: fast length adjustment of short reads to improve genome assemblies, Bioinformatics. 27 (2011) 2957–2963. https://doi.org/10.1093/bioinformatics/btr507.
14. W. Zhou, Y. Li, X. Liu, S. He, J.C. Huang, Comparison of microbial communities in different sulfur-based autotrophic denitrification reactors, Appl. Microbiol. Biotechnol. 101 (2017) 447–453. https://doi.org/10.1007/s00253-016-7912-y.
